# In Vivo Blood Glucose Quantification Using Raman Spectroscopy

**DOI:** 10.1371/journal.pone.0048127

**Published:** 2012-10-25

**Authors:** Jingwei Shao, Manman Lin, Yongqing Li, Xue Li, Junxian Liu, Jianpin Liang, Huilu Yao

**Affiliations:** 1 College of Chemistry and Chemical Engineering, Fuzhou University, Fuzhou, China; 2 Laboratory of Biophysics, Guangxi Academy of Sciences, Nanning, China; 3 College of Physics and Technology, Guangxi Normal University, Guilin, China; 4 Department of Physics, East Carolina University, Greenville, North Carolina, United States of America; University of Zurich, Switzerland

## Abstract

We here propose a novel Raman spectroscopy method that permits the noninvasive measurement of blood glucose concentration. To reduce the effects of the strong background signals produced by surrounding tissue and to obtain the fingerprint Raman lines formed by blood analytes, a laser was focused on the blood in vessels in the skin. The Raman spectra were collected transcutaneously. Characteristic peaks of glucose (1125 cm^-1^) and hemoglobin (1549 cm^-1^) were observed. Hemoglobin concentration served as an internal standard, and the ratio of the peaks that appeared at 1125 cm^-1^ and 1549 cm^-1^ peaks was used to calculate the concentration of blood glucose. We studied three mouse subjects whose blood glucose levels became elevated over a period of 2 hours using a glucose test assay. During the test, 25 Raman spectra were collected transcutaneously and glucose reference values were provided by a blood glucose meter. Results clearly showed the relationship between Raman intensity and concentration. The release curves were approximately linear with a correlation coefficient of 0.91. This noninvasive methodology may be useful for the study of blood glucose *in vivo*.

## Introduction

Diabetes mellitus is rapidly increasing in global incidence. According to the World Health Organization (WHO), there were over 171 million diabetics worldwide in 2000 (2.8% of the population). It is projected that the number of patients will increase to 366 million by 2030 [1]. Diabetes mellitus is a chronic and incurable disease caused by the malfunction of insulin production, which affects the concentration of glucose in the blood [1]. Glucose is the body’s primary energy source, and unregulated blood glucose concentrations can cause many medical complications. Today, diabetes is diagnosed by measurement of blood sugar levels. Diabetics need to supervise their glucose levels closely and measure them several times a day [2].

The current approach uses small blood volumes extracted from the finger, palms, thighs, forearms, or abdomen to measure glucose. Various alternative sensor devices that would allow doctors and patients to measure glucose levels precisely without the discomfort of extracting blood samples have been proposed and implemented [3]. Repeated drawing of blood can induce substantial pain and increase the risk of infection. It also incurs significant costs due to the number of test strips required. In that sense, non-invasive glucose meters represent a major breakthrough [4]. Optical methods have been proposed as a painless and accurate means of glucose measurement [5], [6].

Existing non-invasive optical techniques include near infrared spectroscopy, Raman spectroscopy, photo-acoustic spectroscopy, femtosecond pulse interferometry, optical coherence tomography, and different types of fluorescence [7]–[26]. Common limitations of these approaches include poor glucose specificity and sensitivity and interference from background species. NIR Raman spectroscopy has successful measured glucose at physiologically relevant concentrations in serum, whole blood, and even in interstitial fluid of human volunteers [28]–[31].

However, there is no clinically accurate and robust algorithm that can predict glucose concentrations in multiple human subjects or in the same subject at different times [25]. The physiological lag between blood and interstitial fluid (ISF) glucose is a major challenge for non-invasive measurements of glucose concentration. This is a particular problem with spectroscopic techniques, which predominantly probe ISF glucose, creating inconsistencies in the calibration of methods in which blood glucose is used as a reference [23]. To overcome this problem, many research groups have used arithmetic techniques such as dynamic concentration correction (DDC) and partial least squares (PLS) [23], [27]. However, accuracy remains low for spectra based on combinations of skin heterogeneity and glucose kinetics parameters. Chaiken used tissue-modulated Raman spectroscopy to noninvasively measure blood glucose concentration [31]. In the present study, the measurement sequence consisted of an unpressed and a pressed state. In the unpressed state, the measured Raman spectrum included contributions from both tissue components and blood analytes. In the pressed state, pressure placed on the finger causes much of the blood to leave the irradiated capillary bed. Raman spectra measured under these conditions tend to show lower peaks. The glucose concentration can be calculated using these Raman spectra. In the unpressed state, spectra may be affected by actors such as displacement and blood in the capillaries.

Here, we report a novel Raman spectroscopy method suitable for transcutaneous monitoring of glucose concentration using focused lasers on blood in vessels in the skin to directly assess blood Raman spectra. We determined the background spectra near the vessels. This method can solve the problems mentioned above. We used hemoglobin as an internal standard. The ratio of the height of peaks appearing at 1125 cm^-1^ and at 1549 cm^-1^ was successfully used to determine the glucose concentrations in the blood. The results show that this method can be used for quantitative analysis of blood glucose. It was found to be highly accurate, strongly linear, and reproducible.

## Materials and Methods

### Animal Models

Four-week-old SPF grade Kunming mice weighing 25–35 g, were purchased from Guangxi Medical University. This study was performed in strict accordance with the recommendations of the Guide for the Care and Use of Laboratory Animals of the National Institutes of Health. The protocol was approved by the Guangxi Medical University Institutional Animal Care and Use Committee (Permit Number: 12-215). All experiments were performed under chloral hydrate anesthesia, and every effort was made to minimize suffering.

**Figure 1:**
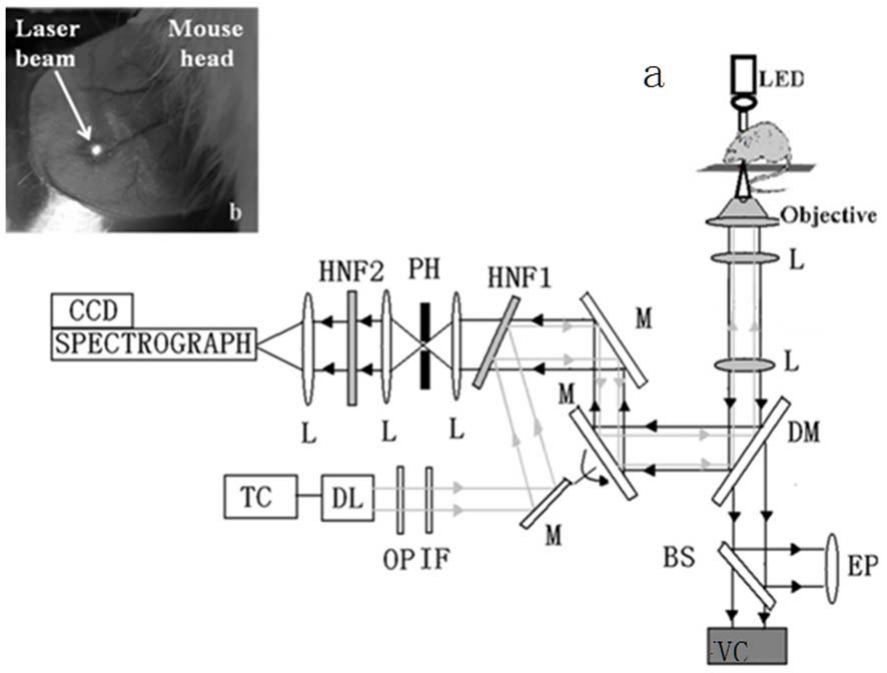
(A) Experimental setup: A laser beam was introduced into a microscope and focused into the selected blood vessels of live mice to collect Raman spectra. Backwards Raman scattering light from blood was projected into the entrance slit of a spectrograph. TC, temperature controller; DL, diode laser; OP, optical isolator; IF, interference filter; M, mirror; L, lens; PH, pinhole; HNF1, HNF2, holographic notch filter; CCD, charge-coupled detector; DM, dichronic mirror; BS, beam splitter; Obj, objective lens; EP, eyepiece; VC, video camera. b: Laser beam in mouse ear.

### Chemicals

D-glucose was purchased from Sigma Aldrich. Milli-Q water was used to prepare the glucose solutions at different concentrations throughout the present study.

**Figure 2:**
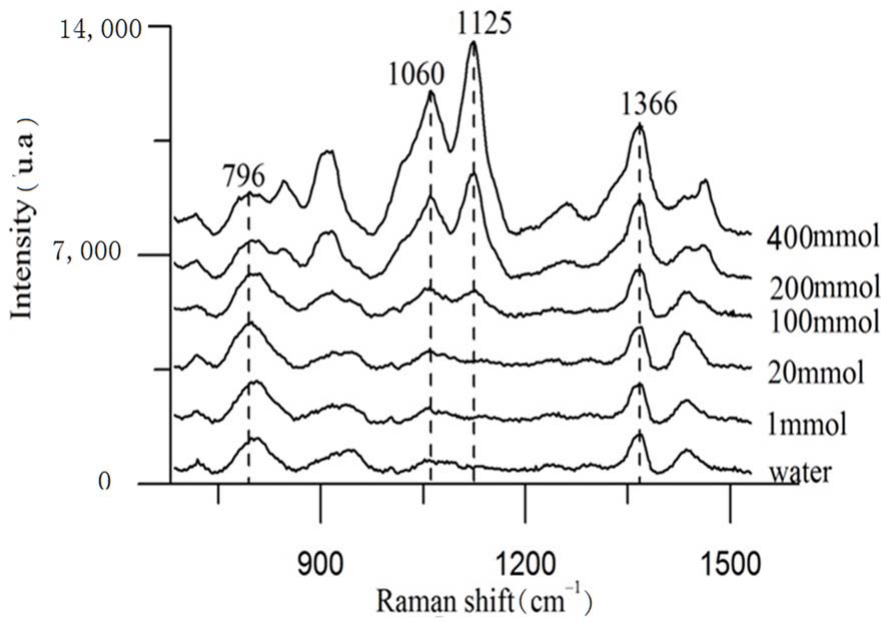
Raman spectra of glucose solutions. The peaks indicative of glucose increase as function of concentration.

### Raman Measurements

The *in vivo* Raman setup employed in this study has been described previously [32]. Fig. 1 depicts the setup. Briefly, a diode laser operating at 785 nm (DL7140, Sanyo) was used for excitation of Raman spectra with an average power of about 15 mW at the sample. The animal subject was anesthetized and placed on the stage with its ear adhered to a microscope slide. The laser beam from a diode laser was introduced into the objective of an inverse Nikon TE-U microscope equipped with a 100×1.3 NA oil immersion objective and focused on the selected blood vessel. The backscattered light was collected using the microscope objective; it first passed through a holographic notch filter and then through a confocal system with a 100 μm pinhole. The spectrometer had grating at 1200 lines/mm and was fitted with a thermoelectrically controlled CCD detector (PIXIS 100 BR, Princeton Instruments), cooled to -120°C. A CCD camera attached to the microscope provided optical images during experiments. Raman spectra were recorded with a spectral resolution of 4 cm^-1^. To determine the locations of the blood vessels to be measured, each ear was transilluminated with a white LED (560 nm) and imaged by the microscope objective lens into a CCD camera.

**Figure 3:**
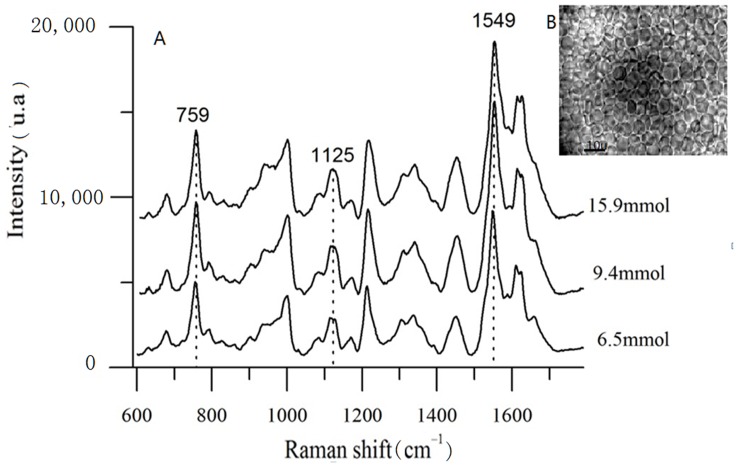
(A) Raman spectra of blood with different glucose concentrations; B: blood.

**Table 1:** Blood glucose concentration and 1125 cm^-1^ relative intensity.

Time (min)	Blood glucose concentration (mmol/dl)	1125 cm^-1^relative intensity
30	4.8	0.240
45	5.6	0.242
60	8.4	0.254
75	12.1	0.258
90	15.9	0.274
105	9.4	0.256
120	7.2	0.252
135	6.5	0.245
150	6.2	0.245

### Sample Preparation

a) *In vitro* experiment: Each mouse was given water *ad libitum* and no food for 24 hours before the experiment. Glucose (5% wt./wt.) was injected into each mouse (0.1 ml/10 g) and blood was taken from the tail 30 min later. Blood samples were collected every 15 minutes. The Raman spectra of blood were taken immediately after blood collection. Raman spectra of each collection were found to contain three spectra. All Raman spectrum acquisition processes lasted 15 s. For reference, blood glucose was also measured using a blood glucose meter (GT-1810, ARKRAY Factory, Inc.).

**Figure 4:**
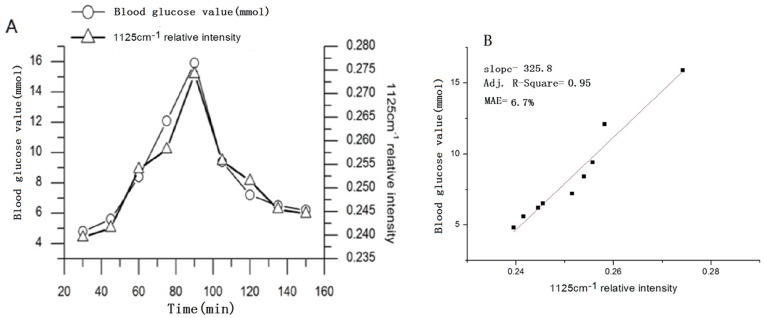
(A) Blood glucose value with 1125 cm^-1^ relative intensity; 4B Concentration-dependent Raman relative intensities of glucose (1125 cm^-1^).

b) *In vivo* experiment: Each mouse was given water *ad libitum* and no food for 24 hours before the experiment, 4% chloral hydrate (0.1 ml/10 g) was injected into the abdomen of mice to anaesthetize them. Glucose (5% wt./wt.) was injected into the anesthetized mice (0.1 ml/10 g). Starting 30 min after the injection of glucose, Raman spectra of the blood in the veins under the skin were collected every 15 min over a 2 h period. Each collection produced three spectra. All Raman spectrum acquisition processes lasted 15 s. Reference blood glucose concentrations were measured from blood taken from the tail using a blood glucose meter (GT-1810, ARKRAY Factory, Inc.). In order to provide data about the reproducibility of the results, three experiments were performed on three separate mice, and 25 data sets were obtained. The background spectra were acquired near the vessels.

**Figure 5:**
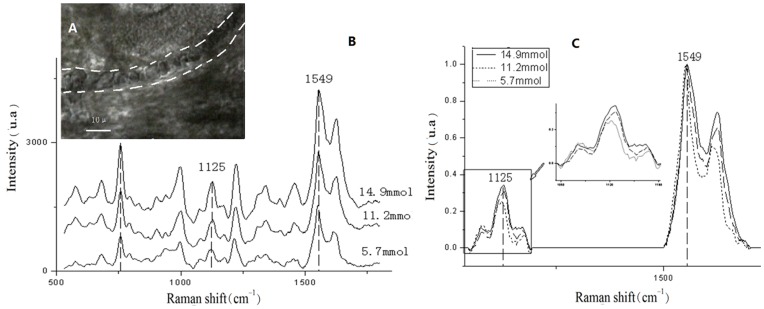
(A) Blood vessel in a mouse ear highlighted by dashed white lines. Scale bar is 10 μm; Fig. 5B: Raman spectra of blood with different glucose concentrations; Fig. 5C Raman spectra after normalization at a height of 1549 cm^-1^.

### Data Analysis

Spectral processing included the removal of cosmic ray spikes, subtraction of background spectra, 5-point adjacent averaging smoothing, baseline calibration, and averaging of the spectra. These actions were performed out using Micro Origin 8.0 software.

**Figure 6:**
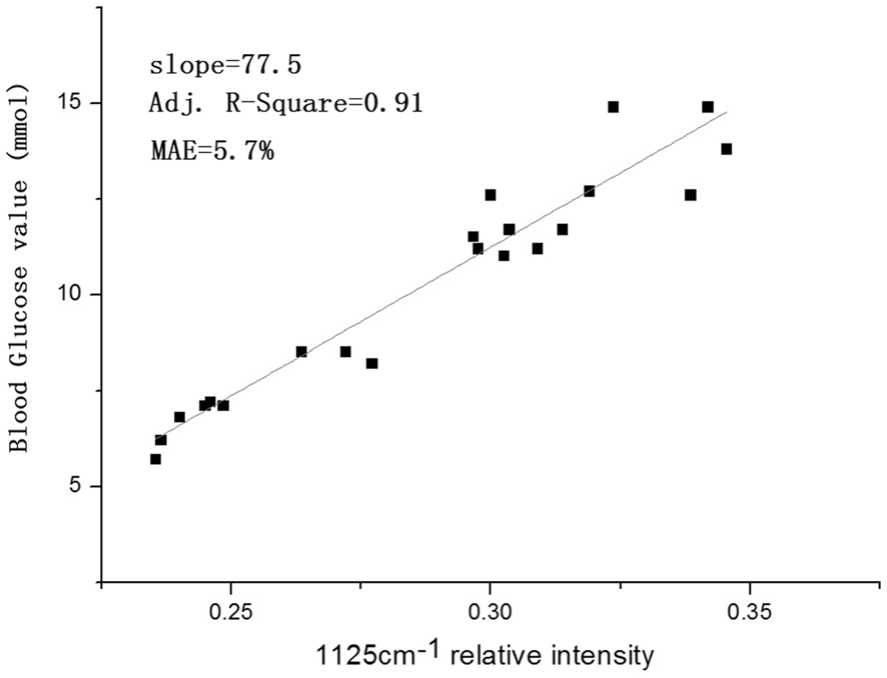
Raman relative intensities of glucose (1125 cm^-1^) in vivo versus the reference values with a mean absolute error of 5.7% and an Adj. R-Square of 0.91.

## Results and Discussion

### Raman Spectra of Glucose Solutions

Fig. 2 shows the Raman spectra of glucose solutions at different glucose concentrations. The spectra show the Raman peaks characteristic of glucose. These values are consistent with the standard spectrum [33]. The major Raman peaks that appeared at 911 cm^-1^, 1060 cm^-1^, and 1125 cm^-1^ are considered the Raman fingerprints of glucose. However, the peak that appeared at 1125 cm^-1^ was the most intense, meaning that it had the best signal to noise ratio. Here, we used only the 1125 cm^-1^ peak to demonstrate the efficacy of this system. The measurements were performed using glucose concentrations of 1–400 mmol/dl, and the intensities of the Raman lines increased as glucose concentration increased. This suggests that the concentration of blood glucose can be determined by measuring its Raman spectrum. However, in practice, the concentration of human blood glucose can be as low as 3–10 mmol/dl for normal individuals and <30 mmol/ml for diabetics. The Raman intensity at very low concentrations is too weak to be measured within a reasonable time. In our study, no Raman signal was detected at glucose concentrations below 50 mmol/dl, even exposure lasted as long as 10 minutes. These results are consistent with those observed in one previous study [34]. We found that changes in Raman intensity could be observed when glucose was present in the blood.

**Table 2:** Linearized curve-fitting parameters of blood glucose concentration and 1125 cm^-1^ relative intensity from three individual mice.

Mouse	Slope	Intercept	Adj.R-S
First	71.8	−9.2	0.89
Second	81.9	−13.1	0.96
Third	79.4	−12.3	0.89

### Raman Spectra of Blood Glucose *in vitro*

As shown in Fig. 3, the intensities of the Raman lines increased as the concentration of glucose increased. Because of the complexity, individuality, and diversity of the tissues surrounding the blood vessels, an internal standard had to be selected to ensure that the correct data were collected. The concentration of hemoglobin is more stable than that of glucose, and intra-species differences in the concentration of hemoglobin are small. For this reason, the concentration of hemoglobin was chosen as internal standard. The relative intensity of glucose was obtained through normalization of the height of characteristic peaks of hemoglobin (1549 cm^-1^). This method can be used to determine the concentration of glucose in the blood. Blood glucose concentrations determined with the glucose meter and 1125 cm^-1^ relative intensity are shown in Table 1.

Concentration-dependent Raman intensities of glucose are plotted in Fig. 4B using 1125 cm^-1^ relative intensity. A clear linear relationship appeared between Raman intensity and concentration with a mean absolute error (MAE) in the validated data of 6.7% and an Adj. R-Square of 0.95. This is critical to the quantitative measurement of blood glucose levels.

### Raman spectra of blood glucose *in vivo*

The blood vessel was clearly visible under the microscope (Fig. 5A). Fig. 5B depicts one of the 25 Raman spectra. Fig. 5C shows the spectra after normalization. Figs. 5B and 5C show that the intensity of the 1125 cm^-1^ peaks increases as glucose concentration increases. In this way, the Raman spectra can indicate changes in the level of glucose in the blood *in vivo*.

Blood glucose concentration values depend on the relative intensity of blood glucose after normalization. The intensity of hemoglobin from 25 Raman spectra from 3 mice is plotted in Fig. 6. A clear linear relationship was observed between the Raman intensity and the concentration. It showed a mean absolute error (MAE) in the validated data of 5.7% and an Adj. R-Square of 0.91. Table 2 shows the linearized curve-fitting parameters of Raman intensity and individual concentration. The values of slope and intercept from different mice were found to be similar to each other and to the combined data. These results indicate good reproducibility.

Many methods of measuring glucose are subject to valid questions about whether glucose itself is actually being measured. With this system, however, the presence of glucose was indicated by a peak at 1125 cm^-1^. This is direct spectral evidence that the measurements were produced in response to active glucose concentrations. Focusing the laser on the blood in the vessels in the skin can decrease background signal from tissues. Although significant scattering and attenuation of the incident light were caused by interactions with the tissue, these effects were addressed using background correction. The concentration of hemoglobin served as internal standard, and it was found to reduce the impact of complexity, individuality, and diversity of both tissues and individuals, improving performance. In defined areas, hemoglobin can be carefully controlled physiologically. In this way, calibration is a key to this type of measurement.

### Conclusion

The Raman spectra of glucose solutions were obtained and examined. The spectra showed Raman peaks characteristic of glucose. These peaks can be observed in the blood. A clear linear relationship (R-squared correlation coefficient of 0.91) was observed between the Raman intensity and glucose concentration. To reduce the effect of the strong background signal produced by the tissue and to determine the fingerprint Raman lines formed by blood analytes, a laser was focused on the blood in the vessels of the skin, and the resulting Raman spectra were collected. Using hemoglobin concentration as an internal standard, the ratio of the height of 1125 cm^-1^ against 1549 cm^-1^ was used to calculate the concentration of blood glucose. The results showed that this method can be used for noninvasive, quantitative analysis of blood glucose in vivo. These measurements were found to be highly accurate, strongly linear, and reproducible. Although the detection limit of this technique requires further improvement before it can meet clinical needs, we expect advances in detector sensitivity and background correction to compensate for these limitations.
